# Systematic literature review of economic studies on nature-based social prescribing for health improvement

**DOI:** 10.1186/s12875-026-03258-w

**Published:** 2026-03-14

**Authors:** Sibylle Puntscher, Igor Stojkov, Marjan Arvandi, Felicitas Kühne, Annette Conrads-Frank, Veronika Papon, Daniela Schmid, Beate Jahn, Erica A. Suzumura, Richard Kimberlee, Matthew Jones, Kaisu H. Pitkälä, Anu H. Jansson, Jill S. Litt, Uwe Siebert, Ursula Rochau

**Affiliations:** 1 Institute of Public Health, Medical Decision Making and Health Technology Assessment, Department of Public Health, Health Services Research and Health Technology Assessment, UMIT TIROL – University for Health Sciences and Technology, Hall in Tirol, Austria; 2 Faculty of Life Sciences, Albstadt-Sigmaringen University, Sigmaringen, Germany; 3 Department of Preventive Medicine, Faculdade de Medicina FMUSP, University of Sao Paulo, São Paulo, Brazil; 4 Faculty of Health and Life Sciences, University of the West of England, Bristol, UK; 5 Centre for Public Health and Wellbeing, University of the West of England, Bristol, UK; 6 Department of General Practice and Primary Healthcare, University of Helsinki, Helsinki, Finland; 7 Unit of Primary Care, Helsinki University Central Hospital, Helsinki, Finland; 8 Barcelona Institute for Global Health (ISGlobal), Barcelona, Spain; 9 Center for Health Decision Science, Departments of Epidemiology and Health Policy & Management, Harvard Chan School of Public Health, Boston, MA USA; 10 Institute for Technology Assessment, Department of Radiology, Massachusetts General Hospital, Harvard Medical School, Boston, MA USA

**Keywords:** Nature-based social prescribing, Systematic review, Health economic analyses, Social return on investment, Well-being, Mental health, Physical health

## Abstract

**Background:**

Nature-based social prescribing (NBSP) is a form of social prescribing that can enhance mental and/or physical health by connecting participants with nature through non-medical community referrals. While evidence suggests potential benefits of NBSP in promoting well-being, its economic impact remains unclear. The purpose of our systematic literature review is to investigate the health-economic impact of NBSP programs.

**Methods:**

We searched six databases in July 2024. Two reviewers independently screened publications for eligibility: (1) NBSP interventions for health improvement among adults (2), partial or complete economic analyses, and (3) peer-reviewed original studies, published in English, Spanish, or German after the year 2000. Eligible data were systematically extracted by two independent authors into an extraction form including standardized information on the publication, population and setting, compared strategies, details of the analytic framework, main results, limitations, and study conclusions. This information was further synthesized in an evidence table and analyzed using a narrative synthesis approach, with results summarized for each study. The quality of the included studies was assessed using the Consensus Health Economic Criteria list. The review followed PRISMA guideline and is registered on PROSPERO (CRD42021286176).

**Results:**

Of the 2,985 hits identified, five studies met the inclusion criteria, all of which were conducted in the United Kingdom (UK) and included people with mild mental and/or physical health issues. The time horizon ranged from twelve weeks to ten years. Three studies conducted a social return on investment (SROI) analysis and reported SROI ratios between 2.6 and 5.1 British pounds (GBP) per GBP invested. One cost-benefit study identified economic returns of 6,000–14,000 GBP per person after one year, with potential returns of up to 8,600 − 24,500 GBP per person after ten years. One cost-utility analysis resulted in costs of 8,600 GBP per quality-adjusted life year gained.

**Conclusions:**

Evidence on the health and economic impacts of NBSP is currently limited. The five identified studies, all based in the UK, generally reported favorable health and economic findings. However, the small number of studies, their methodological limitations, and the exclusive UK focus constrain the generalizability of these conclusions. To better inform healthcare decision-making and to implement NBSP programs worldwide, further research on NBSP interventions and their economic impact is needed. Consequently, future studies should explore international settings, incorporate additional data sources, include control groups, and assess longer follow-up periods.

**Supplementary Information:**

The online version contains supplementary material available at 10.1186/s12875-026-03258-w.

## Introduction

Healthcare systems globally are facing mounting pressures from increasing demand, rising costs, and overstretched social services. Notably, around 20% of primary care consultations are attributed to non-medical concerns, such as loneliness, stress, or social isolation, and impose economic and organizational challenges on healthcare systems [1]. The need to better respond to non-medical issues that traditional clinical approaches may not fully address has driven interest in innovative, cost-effective strategies to support public well-being. One such approach is social prescribing (SP), which seeks to link persons with social needs - seeking support in the healthcare system - with additional, non-medical forms of support within their communities [1, 2]. Referral pathways to SP services vary and may include direct referrals from primary care health professionals and care organizations, self-referral by individuals, indirect referrals with specialized link workers, or a combination of these pathways [3]. The specialized link workers support people by connecting them to non-clinical, community-based services for improving their physical and mental health and well-being [2–4].

The concept of SP has been developing and gaining popularity over the past few decades, particularly in the United Kingdom (UK) and as a key element of the National Health Services’ (NHS) strategy [5, 6]. SP involves linking and referring people with health and social care needs to local services and activities that can support their health and well-being [7]. These SP services include a wide range of activities, such as art therapy, reading groups, exercise classes, volunteering, financial, welfare, or legal advice [8, 9]. The idea of SP has been adopted not only in the UK but also in a growing number of countries in Europe, Asia, and North America, although on a more moderate scale [10–12]. However, since the implementation strongly depends on the national context, the overall impact of SP remains unclear. As Morse et al. state, SP may be able to globally support health and well-being, but stronger international cooperation and coordination are needed to develop implementation guidelines [11]. While SP encompasses a broad range of non-clinical interventions, it does not necessarily include structured engagement with natural environments. One distinct subset of SP that explicitly incorporates exposure to nature is nature-based social prescribing (NBSP). This specialized form of SP is defined by the deliberate integration of natural environments as a core component of the prescribed activity and offers corresponding activities in nature to support people with emotional, social, or physical health needs in a community setting [13]. NBSP combines the beneficial effects of social activities with the positive effects of nature exposure on physical and mental health [14]. Being in nature, or even viewing scenes of nature, can help boost one´s mood, improve health, and increase overall physical activity [15–19].

NBSP typically includes activities in green spaces (e.g., parks, forests, and gardens) and blue spaces (e.g., rivers, lakes, and coastal areas), such as walking groups, gardening clubs, kayaking, and river walks [14, 20]. Epidemiological studies have shown that recreational exposure to nature and outdoor activities can simultaneously improve various health aspects, including anxiety or depression [21, 22], reduce physical and psychological stress [23, 24], and enhance innate immunity [25]. Additionally, exposure to nature is associated with health benefits, such as reduced overall mortality and a reduced incidence of cardiovascular diseases or metabolic diseases [26].

Overall, NBSP increases patient satisfaction and effectively complements traditional healthcare interventions in addressing complex and long-term health problems [27, 28]. The final report on the Green Social Prescribing Project, which supported people with mental health issues mainly from socio-economically deprived areas in the UK, indicated statistically significant positive effects on happiness, life satisfaction, and anxiety after nature-based activities [29]. Menhas et al. conclude that NBSP has a positive effect on mental health outcomes and suggest to incorporate NBSP interventions more strongly as a complement to traditional therapies [28]. A community gardening therapy in the UK was reported to improve and maintain well-being of people with mental health problems also during the COVID-19 pandemic [30]. A study in the UK by Garside et al. also identified a positive, albeit not robust, effect of NBSP for people with mental ill health [31]. To improve NBSP implementation, the authors recommend improving the coordination, the information-sharing mechanism between stakeholders, the capacities of local entities, and the funding of NBSP [31].

SP interventions and the inclusion of local services might also relieve pressure on general practitioners (GP), which is an aspect of great importance for the healthcare budget [27, 28, 32]. Health economic evaluations of programs, such as NBSP, are essential for assessing their cost effectiveness and potential to generate cost savings for healthcare systems. These evaluations are critical for fostering broader acceptance of NBSP among healthcare professionals and decision makers [33, 34]. However, the wide variability in NBSP interventions and assessment methods presents challenges in drawing definitive conclusions. A review by Lynch et al. provided first insights on economic evaluations by summarizing the literature on how people value the access to natural environments [35]. The twelve studies included showed significant heterogeneity among the economic methods and types of interventions analyzed. Nevertheless, Lynch et al. conclude that people acknowledge positive health effects by recreational activities in nature and are willing to pay for accessing and improving the quality of natural environments [35]. A rapid scoping review commissioned by the National Academy for Social Prescribing on social prescribing overall concluded that the economic impact of SP schemes is generally positive across the applied economic evaluation methods [36]. A recent systematic review by Lynch et al. further examined health economic evaluations of general social prescribing interventions published since 2015 [37]. The authors stated that social prescribing interventions often reported favorable economic findings, including positive social return on investment (SROI) for nature-based interventions. However, they concluded that the overall availability of rigorous economic assessments constrains clear conclusions about cost effectiveness and provides insufficient evidence to inform sustainable health-policy decision-making [37].

While existing reviews primarily assessed SP more broadly, this systematic literature review focuses exclusively on NBSP. The aim is to provide a comprehensive overview of health economic evaluations of formal NBSP initiatives, specifically aimed at improving participants’ health and well-being. Through this synthesis, we examine how nature-specific components are conceptualized and incorporated within economic evaluations in the wider SP literature.

## Methods

For our systematic literature review, we searched the databases PubMed/MEDLINE, Embase, International Health Technology Assessment Database (INAHTA), Tufts Medical Center Global Health Cost Effectiveness Analysis (GH CEA) Registry, The National Health Service Economic Evaluation Database (NHS EED), and The American Economic Association Electronic Bibliography (EconLit) (last update July 1, 2024). We also used ‘Connected Papers’, an automated software based on an algorithm of co-citation and co-referencing, to find studies we might have missed with the other sources [38]. The systematic review was conducted following the Preferred Reporting Items for Systematic Reviews and Meta-Analyses (PRISMA) guideline [39]. The study protocol was registered in the International Prospective Register of Systematic Reviews (PROSPERO 2021 CRD42021286176). Although the PROSPERO protocol specified to standardize costs using currency conversion, inflation adjustment, and purchasing power parity, this step was not conducted because all included studies were from the UK and reported costs in GBP.

Our study was conducted as part of the EU Horizon 2020 project RECETAS (Re-imagining Environments for Connection and Engagement: Testing Actions for Social Prescribing in Natural Spaces) [12, 40]. The aim of this international consortium research project is to determine the effectiveness and cost effectiveness of the NBSP intervention (Friends in Nature) applied to reduce loneliness and increase health-related quality of life (HRQoL) of people experiencing loneliness.

We included all primary, peer-reviewed studies that conducted partial or full economic analyses of NBSP interventions aimed at health improvement in adults. To capture the full range of economic insights, we consider all types of health economic analyses, such as cost-effectiveness analysis (CEA), which evaluates costs relative to specific health outcomes; cost-utility analysis (CUA), which assesses cost per quality-adjusted life year (QALY) gained; cost-benefit analysis (CBA), which compares costs and benefits in monetary terms; and social return on investment (SROI), which estimates broader social, environmental, and economic value created per unit of investment [41, 42]. Given the substantial methodological heterogeneity across these economic evaluation types, findings were synthesized narratively, with results organized by individual study, and no direct quantitative comparisons across studies were conducted. No formal synthesis of effectiveness was performed. We also considered relevant review studies as potential sources for further economic studies. We excluded grey literature to ensure the inclusion of peer-reviewed studies only and studies on environmental goods (e.g., biking or walking trails, swimming pools, marine usage) without an explicit and central component of NBSP, which is accompanied by an economic analysis. Studies had to be published in English, German, or Spanish after the year 2000. Further details on the selection process and search code are presented in the Supporting Information.

Titles and abstracts were screened against inclusion criteria independently by two out of a pool of four reviewers (IS, MA, VP, SP). To ensure a consistent approach to study selection, a brief screening guidance document (see Supplementary Information) was developed and discussed with the reviewers involved in the screening process in advance. Full text copies of articles meeting initial screening criteria based on abstract/ titles were retrieved for further assessment against inclusion criteria by two reviewers independently (IS, MA, VP, SP). Disagreements between reviewers regarding inclusion of articles at any stage of screening were resolved through discussion until consensus was reached. If consensus could not be reached, an additional reviewer was consulted and made the final decision (UR). Data were extracted independently by two out of a pool of four reviewers (IS, MA, VP, SP) from the original sources using a pre-specified extraction form. The extraction form recorded information on study context and intervention characteristics, as well as key economic evaluation domains, including evaluation type, perspective, time horizon, costing approach, outcomes, results, reported limitations and authors’ conclusions. The selection of extraction domains was informed by established health economic reporting standards, including the Consolidated Health Economic Evaluation Report Standards (CHEERS) guidelines [43].

We conducted the quality assessment for all included studies independently using criteria based on the Consensus Health Economic Criteria (CHEC) list [44]. The CHEC list was chosen as a widely used, validated, and comprehensive checklist for the assessment of health economic evaluations. The CHEC list was developed for systematic reviews focusing on full economic evaluations on clinical trials comparing costs and consequences of alternative interventions [45]. As Evers et al. [44] state, the list should be seen as a minimum set for assessing the methodological quality of an economic evaluation. The CHEC list includes 19 items, which can be answered with “yes” or “no” [44]. Following the assessment instructions, the assessor should answer yes, if the study evaluated the aspect under consideration adequately, and no, if the aspect was not discussed sufficiently or if the information given was insufficient [46]. No summary score is calculated based on the CHEC list, as this is not intended and also not recommended in order to avoid oversimplification of the quality assessment [45]. Following the recommendations, the quality assessment was conducted by two reviewers (SP, IS, VP) independently, and disagreements were resolved by discussion and consensus.

For comparability, the information collected and extracted from the original studies was synthesized in a systematic evidence table, including standardized information on the publication, population and setting, compared strategies, details of the analytic framework, main results, limitations, and study conclusions. In addition to the synoptic evidence table, key contents of each individual study were briefly summarized narratively in a systematically structured manner.

## Results

**Fig. 1:**
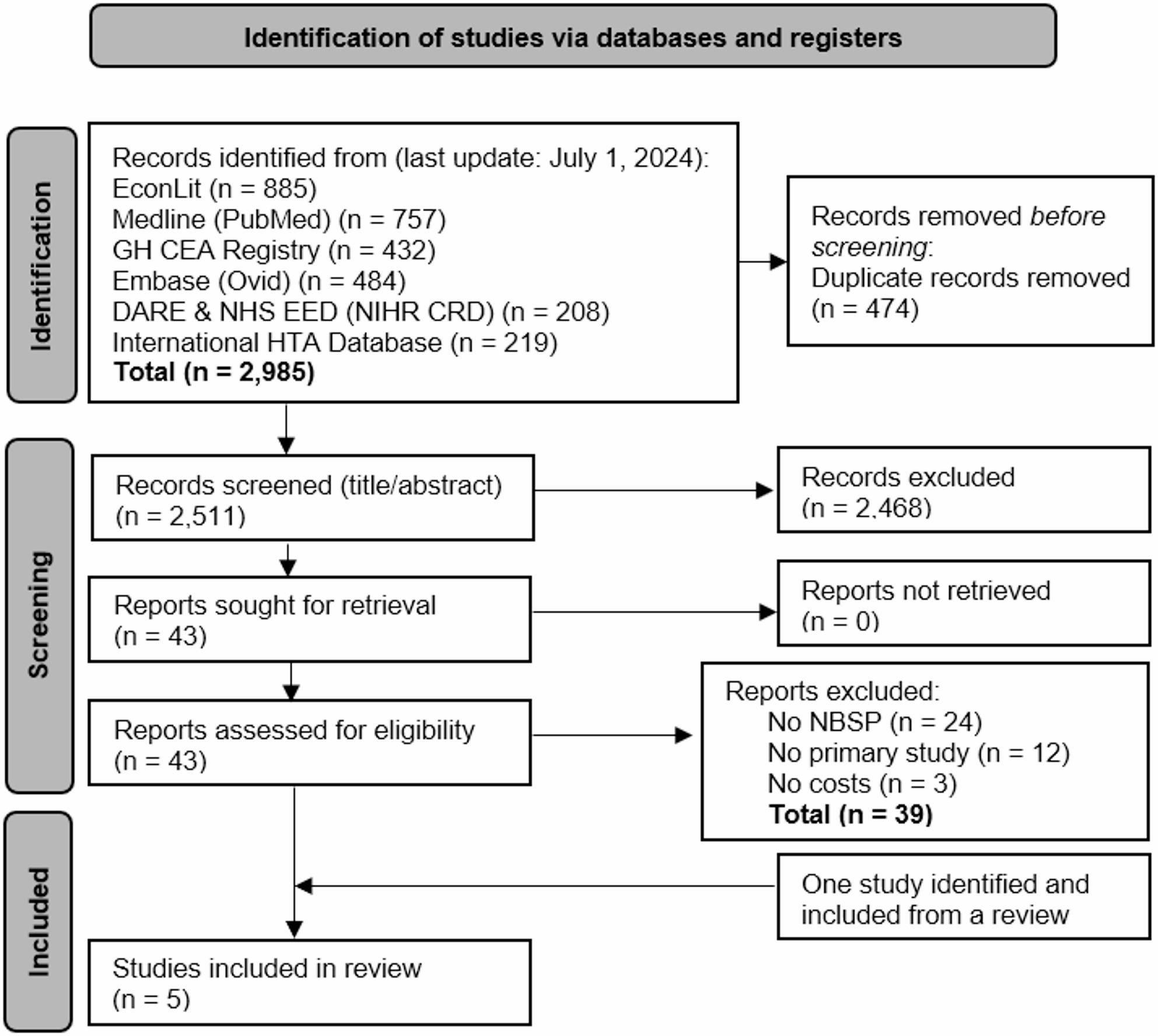
PRISMA 2020 flow chart for the systematic literature review of NBSP economic studies Note: DARE - Database of Abstracts of Reviews of Effects; EconLit - The American Economic Association Electronic Bibliography; GH CEA - The Global Health Cost Effectiveness Analysis Registry; HTA - Health Technology Assessment; NBSP - Nature-based social prescribing; n – number; NHS EED - The National Health Service Economic Evaluation Database; NIHR CRD - The National Institute for Health Research Centre for Reviews and Dissemination - Centre for Reviews and Dissemination. The flow diagram is adapted based on the updated version of the Preferred Reporting Items for Systematic Reviews and Meta-Analyses (PRISMA 2020) Statement [52]

In total, we identified 2,985 records from our search, with 2,511 remaining after duplicate removal. After title and abstract screening, 43 studies were included for full-text screening. One additional study [47] was identified from a published review [35]. As shown in the PRISMA 2020 flow chart in Fig. 1, five studies fulfilled the inclusion criteria [47–51]. Their content was extracted and summarized in the systematic evidence table (Table 1). No additional relevant studies were identified with the software ‘Connected Papers’.

**Table 1:** Evidence table from the NBSP economic studies literature review

Characteristics	Hartfiel et al. [50]	Jones et al. [49]	Makanjuola et al. [51]	Pretty et al. [48]	Willis et al. [47]
Country	Wales, UK	Wales, UK	Wales, UK	England, UK	Scotland, UK
Health program evaluated	Coed Lleol – Small Woods Wales	Health Precinct Community Hub	Opening doors to the outdoors (ODO) program	NBIs/MBIs interventions	Branching Out program
Population/setting	People with mild mental health challenges / adults experiencing mental or physical health challenges or both	Individuals aged 55 and older, with chronic conditions, with mild or without cognitive impairment living at home (no residential care) and had at least one consultation within the Health Precinct	Adults experiencing a physical, mental or social issue	Adults and young people facing different challenges (e.g., mental health issues, special educational needs, substance misuse, veterans, refugees, marginalized groups)	Adults experiencing severe and enduring mental health problems in low- or medium-secure rehabilitative care.
Intervention(s)	Weekly 2–4 h sessions: (1) multi-activity program; (2) mindfulness-in-the-woods pilot program	Treatment plan including bowling, boating lake, tennis courts, occupational therapy, nursing advice, physiotherapy	ODO program with a weekly 4-hour session: (1) outdoor walking group, (2) climbing group incl. social sessions (picnic, cafe visit)	(1) Green Light Trust (NBI); (2) Ecominds (NBI); (3) Trust Links Growing Together (NBI); (4) Living Movement Tai Chi (MBI)	Nature conservation, environmental art, green exercise, outdoor skills
Intervention duration	1) 8–12 weeks 2) 6 weeks	16 weeks	12 weeks	Variable	12 weeks
Comparators	No control group Pre-post comparison	No control group Pre-post comparison	No control group Pre-post comparison	No control group Pre-post comparison	No control group Pre-post comparison
Outcomes	Mental well-being (SWEMWS); Physical activity (7-day PA recall question); Self efficacy (General Self-Efficacy Scale); Social trust	Physical activity; Health status (EQ-5D-5 L); Confidence level (Rosenberg self-esteem scale); Social connection (Campaign to End Loneliness Scale); Health/social care resource use; Health outcomes from family members	Mental well-being (SWEMWS); Social trust; Subjective Health; Physical activity (International Physical Activity Questionnaire)	Loneliness reduction; Life satisfaction/Happiness	QALYs (SF-6D instrument)
Type of evaluation	SROI analysis	SROI analysis	SROI analysis	CBA analyses	CUA analysis
Time horizon	6 months	1 year	12 weeks	Varying period from 1 to 10 years	1 year
Perspective	Not clearly stated (only program costs included)	Societal	Not clearly stated (only program costs included)	Not clearly stated	Not clearly stated
Currency	GBP	GBP	GBP	GBP	GBP
Cost year	Not clearly stated	2018	Not clearly stated	2016–2020	2007/2018 & 2011/2012
Discounting	Not clearly stated	N/A	Not clearly stated	3.5% discount rate; 2% GDP deflator	N/A
Results	Main analysis: SROI ratios 2.57–4.67:1 GBP Sensitivity analyses: SROI ratios 1.11–4.67:1 GBP	Main analysis: SROI ratio 5.07:1 GBP Sensitivity analyses: SROI ratios 2.60:1 GBP − 5.16:1 GBP	Main analysis: SROI ratios 4.90–5.36:1 GBP Sensitivity analyses: SROI ratios 4.37–5.36:1 GBP	*CBA*: Total economic benefits: 6,144–14,332 GBP per person at year 1; 8,602–24,568 GBP per person at year 10. *Public benefits–private costs ratio at year 1*: GLT: 15.1:1 GBP Trust Links: 6.42:1 GBP	8,600 GBP per QALY (participating in at least one session) 11,480 GBP per QALY (proportional to the number of sessions)
Deadweight, displacement, attribution, attrition	Questions to participants and standard percentages: deadweight = 27%, displacement = 5% attribution = 46%,	Questions included in the follow-up for participants: deadweight = 25%, displacement = 0, attribution = 50%, and attrition = 50%	Questions included in the follow-up for participants: deadweight = 49%, displacement = 17%, and attribution = 47% (27% standard deadweight% applied for health outcomes when using the Social Value Calculator)	N/A	N/A
Limitations	*Limitations mentioned*: - no control group; - other factors (e.g., weather) not considered; - researcher bias and the likelihood that estimates of social value can be upward biased; - Coed Lleol—Small Woods Wales multi-activity program is not standardized	*Methodological limitations* - lack of equity weightings, - variation in methods used to value outcomes, - no guidance on interpretation of SROI values *Data limitation*: - family member data available only for 38/66 participants completing the program	*Limitations mentioned*: - case study approach; - small sample size; - no control group; - no access to participants’ medical records, data depended on participants’ recall of mental health service use over the period investigated; - other influences (e.g., weather) not considered	*Limitations mentioned*: -no preventative policies adopted at a scale that can be evaluated; -differing levels of biodiversity and landscape quality type not considered; -not possible to disentangle the effects of different program components (e.g., physical activity, cognitive engagement, presence of nature, healthy food)	No limitations mentioned
Conclusion	“The results showed that Coed Lleol—Small Woods Wales programmes generated positive social value for participants. Quantitative data from baseline and follow-up questionnaires indicated that many participants improved in mental wellbeing, physical activity, self-efficacy, and social trust.”	“The amount of social value generated by the Health Precinct outweighed the cost of inputs required to deliver their programmes.”	“Overall, our analysis showed that ODO programmes generated positive social value ratios ranging from £4.37 to £5.36 for every £1 invested. Quantitative and qualitative data from baseline and follow-up questionnaires indicated that many ODO participants improved in mental wellbeing, physical activity, social trust, and overall good health.”	“NBIs and MBIs can play a role in helping to reduce the costs on health systems, while increasing the well-being of people with mental health issues.”	“The Branching Out programme leads to a QALY improvement in the short term. If QALY improvements last one year, then the value per QALY would be £8600, which is good value for money in terms of NICE guidelines; however, if QALY improvements only last three months, this would indicate a value of £34,343 per QALY, marginally above the NICE guidelines.”
CHEC list assessment (items out of 19)	Yes: 12 No: 3 N/A: 4	Yes: 13 No: 3 N/A: 3	Yes: 12 No: 3 N/A: 4	Yes: 9 No: 7 N/A: 3	Yes: 11 No: 4 N/A: 4

*CBA* Cost-benefit analysis, *CEA* Cost-effectiveness analysis, *GBP [£]* British Pound, *HRQoL* Health-related quality of life, *LS/H* Life satisfaction/happiness, *MBI* Mind-body interventions, *N/A* Not applicable, *NBI* Nature-based interventions, *NBSP *Nature-based social prescribing, *NICE* National Institute for Health and Care Excellence, *ODO* ‘Opening Doors to the Outdoors’, *QALY* Quality-adjusted life year, *SROI* Social Return on Investment, *SWEMWS* Short Warwick Edinburgh Mental Wellbeing Scale, *UK *United Kingdom

All five included studies were conducted in the UK, with three of the studies situated in Wales [49–51], one in Scotland [47], and one in England [48]. Despite variations in the study populations, individuals with mild mental health issues were part of the study population in all five studies, reflecting a common focus on mental well-being-oriented NBSP. Across studies, participant recruitment was based on self-referral and/or referral by healthcare, social care, or community professionals, with participation being voluntary in all cases. The intervention durations were six to twelve weeks [50], twelve weeks [47, 51], 16 weeks [49], or without a fixed timeframe [48]. The analytic time horizons applied in the economic evaluation varied considerably, ranging from three months [51] to ten years [48]. An explicit analytic perspective was reported in only one study [49], which adopted a societal perspective. The remaining studies did not provide a clear perspective, but it might be implied based on the types of costs and outcomes included: Pretty et al. might reflect a public sector perspective [48], Willis et al. adopted rather a public sector service provider perspective [47], and Hartfiel et al. and Makanjuola et al. seem to adopt a stakeholder-focused perspective for their respective SROI studies [50, 51].

None of the included studies incorporated a control or comparison group and relied exclusively on pre-post comparisons. The economic analyses considered non-market outcomes, including improvements in mental well-being, social connectedness, and quality of life. Despite heterogeneity in study design, intervention type, population, and valuation approaches, all studies reported favorable economic findings, indicating that estimated benefits exceeded associated costs under the assumptions applied. Methodological variability was evident in outcome measurement, valuation assumptions, and time horizons. These overarching patterns provide important context for the individual study findings presented below.

Given the methodological heterogeneity, the included studies are individually described in alphabetical order in the following sections, based on the data summarized in the systematic evidence table (Table 1), with detailed results of the CHEC list-based quality assessment provided in Table S1 in the Supporting Information. For each study, we describe several extracted key factors from Table 1, including the description of the NBSP intervention, the targeted study population, the study sample, type of health economic analyses applied, outcomes assessed, costs considered, results and the corresponding authors’ interpretation, followed by the limitations as mentioned by the authors.

### Hartfiel et al. [50]

Hartfiel et al. [50] conducted SROI analyses based on data from 120 individuals participating in Coed Lleol - Small Woods Wales programs between 2017 and 2019. The eligible participants were adults with mental and/or physical health issues. The multi-activity programs ran over eight to twelve weeks and were organized as weekly sessions, including physical activity and either nature-based or craft-based activities over two to four hours. The mindfulness-in-the-woods program was a six-week pilot intervention based on the idea of an existing stress reduction program [50]. The participants answered a questionnaire at baseline and at follow-up, allowing a pre-post analysis but no comparison with a control group. The main outcome of the study was mental well-being measured with the Short Warwick–Edinburgh Mental Wellbeing Scale (SWEMWBS). Physical activity using the 7-Day Physical Activity Recall Question, self-efficacy using the General Self-Efficacy Scale, and social trust based on the New Economics Foundation Social Trust Question were mentioned as secondary outcomes. The outcomes were valued for the SROI analyses applying the Mental Health Social Value Calculator for the primary outcome and the Social Value Calculator for the secondary outcomes. The costs considered included the costs for organization of the program (e.g., staff, coordination of referrals) and the costs for the sessions (e.g., transportation, information material). No information is provided on discounting or inflation adjustments. Following the methodology of a SROI, the study considers deadweight (27%), attribution (46%), and displacement (5%) [50]. Deadweight refers to outcomes that would have occurred without the intervention, attribution measures the intervention’s direct contribution to outcomes, and displacement occurs when the benefits of the intervention shift from one group to another rather than generating new value [53].

The study concluded that “GBP 2.57 to GBP 4.67 of social value was generated for every GBP invested in Coed Lleol—Small Woods Wales programmes” [50]. The authors argue that the results indicate a positive social value supporting the possibility to improve mental well-being, which were comparable to other NBI. Still, the long-term effects of such initiatives and the actual change in the use of health and social care resources are subject to future research. 

The limitations mentioned by the authors in this study include the absence of a control group, the failure to account for external factors, such as weather, and the potential for researcher bias, which could lead to upwardly biased estimates of social value. Additionally, the Coed Lleol—Small Woods Wales multi-activity program was not standardized, which may affect the generalizability of the findings [50].

As reflected in the CHEC list assessment, interpretation of the (favorable) SROI results is influenced by methodological limitations, including the absence of a control group, a short and insufficiently justified time horizon, and the lack of sensitivity analyses to address uncertainty in key valuation assumptions, despite comprehensive identification, measurement, and valuation of costs and outcomes [50].

### Jones et al. [49]

Jones et al. [49] conducted SROI analyses of the 16-week Health Precinct program. The participants were older than 55 years, living at home, and with no or only mild cognitive impairment. The study examines changes from before to after the intervention and, therefore, did not include a comparison with a control group. Data were collected between October 2017 and September 2019 from 159 participants at time point of referral (baseline) and after four months [49]. The main outcomes on health and well-being were collected using the EQ-5D-5 L questionnaire for HRQoL, the Campaign to End Loneliness Scale, and the Rosenberg Self-Esteem Scale together with questions about demographics, health status, and health and care resource use. The costs considered for the SROI analyses included costs for GP appointments based on national data and the program costs. These were collected from the program administrator and included attendance fees, staff costs, overheads, and equipment costs. An annuitization over twelve years with a discount rate of 3.5% was considered for the equipment costs. For the monetization of the outcomes, the Social Value Calculator was used. Based on this information, the base case SROI analysis resulted in a ratio of 5.07 GBP in benefits for one GBP invested, assessed by using the abovementioned costs and health-related outcomes, such as physical activities, health status, confidence level, and social connection. More SROI analyses were conducted as sensitivity analyses and resulted in ratios between 2.60 : 1 GBP and 5.16 : 1 GBP. Overall, the study concludes that the social value was greater than the associated costs and that the “value was generated to people who were prescribed to the programme, their families, the NHS, and the local government” [49].

The limitations identified in this study include the lack of equity weightings in the economic analysis, the variation in methods used to value outcomes, and the absence of clear guidance on interpreting SROI ratios [49]. Additionally, data limitations were noted, as family member data were available only for 38 out of the 66 participants who completed the program, potentially limiting the comprehensiveness of the findings [49].

According to the quality assessment, the economic results reported by Jones et al. should be interpreted with some caution due to the lack of sensitivity analyses, limited discussion of generalizability, and insufficient consideration of ethical and distributional issues, despite otherwise comprehensive reporting of the study population, analytic perspective, time horizon, and cost identification [49].

### Makanjuola et al. [51]

Makanjuola et al. [51] evaluated a nature-based twelve-week intervention program called Opening the Doors to the Outdoors (ODO) organized in Wales applying a SROI analysis. The target population of the program are adults with poor mental or physical health and an inactive, sedentary lifestyle [51]. Participants were assigned either to the outdoor walking or the climbing group and met weekly for four hours. The weekly sessions do not only focus on outdoor activities but also on socialization of the participants. Outcome data on mental well-being (SWEMWBS), social trust, overall health, and physical activity (International Physical Activity Questionnaire) was collected in interviews using validated questionnaires from 52 participants between April and November 2022 at baseline and follow-up. The monetary value of the outcomes was derived using the Social Value Calculator (for trust, health, physical activity) and the Mental Health Social Value Calculator (for mental well-being). The intervention costs considered included website maintenance, equipment, overheads, staff, instructors, expenses for café visits or picnics (socialization), admission fees, and transport. The total annual costs for the ODO program are calculated to be 74,129 GBP (706 GBP per participant, based on 105 yearly ODO participants). Deadweight (49%), attribution (47%), and displacement (17%) were considered for the calculation of the social value of the outcomes trust, overall health, and physical activity, and resulted in an overall social value of 134,653 GBP or of 3,458 GBP per participant. The social value of mental health, using a standard deadweight of 27%, amounts to 3,788 GBP per ODO participant (*n* = 51). The SROI ratios calculated by the authors as the social value of ODO participant outcomes divided by the costs of the ODO program are indicated to range between 4.37 : 1 GBP and 5.36 : 1 GBP invested. Thus, the authors conclude that the ODO programs generate good value for the investment. This is supported also by qualitative interviews conducted with the participants on the changes in the outcomes. Nevertheless, the authors also indicate that a change in the actual use of healthcare resources has not been assessed and that the considered time period observed should be longer for understanding whether the positive effects are sustainable [51].

The study’s limitations identified by the authors include its case-study approach, small sample size, and the absence of a control group. Furthermore, there was no access to participants’ medical records, and the data relied on participants’ recall of mental health service use during the study period, which could introduce recall bias. Additionally, other external influences, such as weather, were not considered, potentially confounding the results [51].

In line with the CHEC list assessment, the SROI findings of Makanjuola et al. are based on well-defined populations and appropriately measured and valued costs and outcomes. However, interpretation is limited by a short time horizon, lack of sensitivity analyses, and insufficient discussion of ethical and distributional aspects [51].

### Pretty et al. [48]

The study by Pretty et al. [48] evaluated the effect of one mind–body (tai chi) and three nature-based (woodland therapy, community gardening, ecotherapy) intervention programs with different length. The study conducted cost-benefit analyses and calculated additionally a public benefit-private costs ratio for the woodland therapy and the gardening initiative only, for which more detailed data on prevented public health and services costs were available. No comparisons with control groups were conducted, however, some findings were discussed using UK averages and UK proportions as benchmark [48]. The participants of the four programs were people from vulnerable subgroups, adults with mental health problems or learning disabilities, as well as people from the general population. In total, data from 642 participants included in the four programs were gathered from existing databases (woodlands: 2019–2020; gardening: 2017–2020; ecotherapy: 2013; tai chi: 2016–2018). The main outcome was life satisfaction/ happiness (LS/H) measured on a 10-point Likert Scale following the Office for National Statistics of the UK and the World Happiness Reports [54]. The monetary valuation of these outcomes is based on the reduced use of public health services, on reduced health costs due to lower levels of loneliness, and on benefits created from improved happiness measured as income equivalent. These benefits are compared with the costs of intervention and delivery in the programs.

The costs of interventions included in the analyses were collected from the program manager or published literature and are reported to vary between 320 GBP and 1,400 GBP [48]. Pretty et al. [48] concluded that participation in the intervention programs significantly increased overall levels of LS/H among participants. Considering an annual discount rate of 3.5% and an annual inflation rate of 2%, the study identified total economic benefits per person between 6,144 GBP and 14,332 GBP after one year, reaching up to between 8,602 GBP and 24,568 GBP per person after ten years. The additionally calculated public benefit-private cost ratio including all benefits for the woodland program (Green Light Trust) varied between 1.71 and 2.47 after one year and between 12.9 and 19.1 after ten years if considering prevented costs only [48]. The ratio rises to 15.1–15.8 after one year and 15.8–27.1 after ten years if total benefits (i.e., including the effect on life satisfaction) are considered. The gardening program (Trust Links Growing Together) identified a benefits-to-costs ratio for total benefits of 6.42 after one year and of 7.61 after ten years. Thus, the authors conclude that “these social return on investments in the GLT NBIs [Green Light Trust nature-based intervention] are favourable compared with other NBI and SP programmes (typically 2.0–5.0 to 1.0), and highly beneficial when compared with no intervention” [48]. Overall, Pretty et al. concluded that the considered NBI programs can effectively improve LS/H of the participants, but that the long-term effects are currently unclear and that the success of these programs does “not become a reason for cutting public services” [48].

The study acknowledges certain limitations, notably the absence of large-scale preventative policies suitable for evaluation, as well as the failure to account for variations in biodiversity and landscape quality. Additionally, it was not possible to isolate the specific effects of different program components, such as physical activity, cognitive engagement, exposure to nature, and healthy food [48].

Based on the quality assessment, the economic findings reported by Pretty et al. are supported by an appropriate study design and time horizon; however, interpretation is limited by incomplete reporting of the study population and analytic perspective, incomplete identification of relevant costs and outcomes, the absence of sensitivity analyses, and insufficient discussion of ethical and distributional aspects [48].

### Willis et al. [47]

Willis et al. [47] conducted cost-utility analyses of the twelve-week woodland-based voluntary NBSP program called Branching Out, organized mainly for patients with severe and chronic mental health issues in rehabilitative care in Scotland. The program engages patients in weekly three-hour sessions of group-based activities held in a woodland environment, offering opportunities for social interaction, physical exercise, and recreation. These sessions take place in a community-like outdoor setting and involve collaboration between therapists, service coordinators, and participants [47]. The dataset included mainly data from participants surveyed in 2011/2012 (*n* = 73) who completed both the pre-questionnaire at baseline and the post-questionnaire three months after the intervention. The data on HRQoL was compared to the corresponding data of participants at the Branching Out program of 2007/2008 (*n* = 77) collected by another study [55]. The CUA of Willis et al. was, however, based only on the 2011/2012 cohort. For calculating the costs per person in the 2011/2012 survey, the authors used the number of participants who actually attended at least one session of the program and were consequently defined as “potential beneficiaries” (*n* = 335) [47].

The analyses compared the situation before and after the intervention, without comparison with a control group. HRQoL was measured using the SF-12 before and after participation in the twelve-week program. The QALYs were calculated based on the corresponding SF-6D questions. The mean QALY change for the 2011/2012 period of 0.0495 (standard deviation = 0.105; *n* = 73) indicates a significant improvement from before to after the intervention. The total costs considered for the CUA included costs for staff, delivery agents, set-up, referring agencies, and travel, and sum up to overall 426 GBP per program user (*n* = 335). Based on these costs per person, Willis et al. concluded that the before-after cost-utility ratio of the program amounts to 8,600 GBP costs per QALY for participants completing the questionnaires at both time points (*n* = 73) and to 11,460 GBP per QALY for all participants (*n* = 335). The authors consequently identified the program to be cost effective in comparison to the usually suggested threshold of the National Institute for Health and Care Excellence (NICE) in the UK of 30,000 GBP per QALY [56, 57]. This evaluation of the cost effectiveness depends, however, on the duration of the health improvement after the outdoor activity stops. Willis et al. did not report any limitations of their study [47].

Following the quality assessment items, the economic analysis by Willis et al. is supported by a clearly defined research question, an appropriate study design, and comprehensive cost measurement and valuation, but is limited by missing sensitivity analyses, limited discussion of generalizability, and insufficient consideration of ethical and distributional aspects [47].

### Quality assessment

The quality assessment was conducted based on the CHEC list [44] and is summarized in Table S1 in the Supporting Information. Across studies, several methodological strengths were observed, including clearly defined research questions (item 3), appropriate study designs (item, 4), comprehensive identification and valuation of costs (item 7–9) and outcomes (item 10–12), conclusions that followed from the data reported (item 16), and clear statements indicating the absence of conflicts of interest (item 18). However, recurring limitations were also evident. Most studies did not conduct sensitivity analyses to explore uncertainty in key assumptions (item 15), and ethical and distributional considerations were not discussed appropriately (item 19). In addition, issues related to time horizon selection (item 5) and generalizability (item 17) were not addressed in two out of five studies. These issues are relevant when interpreting the generally favorable economic findings reported across the included studies.

## Discussion

Our systematic review identified and summarized economic evaluations on existing SP programs with a specific nature-based focus. We identified five studies comparing costs and benefits of NBSP interventions based on SROI [49–51], a CBA [48], and a CUA [47] with results generally indicating positive economic and well-being outcomes associated with NBSP.

This aligns with the broader literature on economic evaluations of general SP interventions, without a specific focus on nature-based approaches, which have reported mostly positive results. Bickerdike et al. [2] focused in a systematic review on the evidence for SP programs in the UK and found 15 evaluations in which healthcare professionals refer patients from primary care to a facilitator of SP. Considering health and well-being as primary outcomes, the included studies stated mainly positive conclusions. However, the systematic research identified only weak evidence for the effectiveness of social prescribing and limited evidence of economic value, largely due to methodological shortcomings of the underlying effectiveness studies, including small-scale and poorly reported studies, frequent absence of comparator groups, short follow-up durations, limited use of standardized outcome measures, and limited adjustment for confounding factors [2]. Similarly, the recent review by Lynch et al. [37] stated that the overall evidence base of economic evaluation of social prescribing remains limited. The authors highlighted persistent limitations, including heterogeneous evaluation approaches, limited application of standard health economic methods, and weaknesses in outcome measurement and reporting, which constrain the usefulness of existing evidence for healthcare planning and decision-making [37]. In addition, a pilot intervention study in Wales analyzed more specifically the economic effect of SP with a particular focus on frequent attenders in primary care (i.e., 15 or more GP visits per year) [58]. A potential for cost saving was suggested due to a reduced annual usage of healthcare units. Similarly, a SROI analysis on a pilot SP program on lifestyle coaching in South Wales indicated positive social value ratios for the intervention [59].

As shown by our systematic review, assessments of the economic impact of NBSP interventions are, however, still rare. The five studies evaluating NBSP interventions identified in our systematic review have all been conducted in the UK [47–51]. Thus, the applicability and transferability of the economic findings to other settings and countries may be constrained by differences in health system organization, cost structures, and valuation frameworks. The target populations of all studies included adults with mental and/or physical health issues. Jones et al. and Willis et al. additionally included people with chronic or enduring conditions [47, 49], and one intervention of Pretty et al. additionally included adults and young people with special needs, substance misuse, marginalized groups, refugees, or veterans [48]. None of the studies included in our review considered a control group for comparison but applied before-after approaches for assessing the economic effect. The lack of a control group represents a significant limitation, as it does not reliably allow to draw causal conclusions on the relationship between intervention and observed economic outcomes. Pre-post assessments are susceptible to various biases, such as changes over time unrelated to the intervention, or participant expectation effects, such as the Hawthorne effect, where individuals alter their behavior simply because they are aware of being observed or involved in a study [60, 61]. Without a comparator group, it is challenging to distinguish the true causal impact of NBSP from other confounding external factors, thereby reducing the internal validity of the findings.

The five studies included in our review employed a range of economic evaluation methodologies to assess the impact of NBSP. The SROI approach applied by Jones et al. [49], Hartfiel et al. [50], and Makanjuola et al. [51] goes beyond the traditional return-on-investment methodology by considering social, environmental, and economic impacts in addition to the financial effects of an investment. Impacts without a clear internal monetary value or specifiable market price (e.g., happiness, friendship) are monetized [41]. The SROI analysis allows researchers the identification of health and non-health outcomes, and “analysing and computing views of multiple stakeholders in a singular monetary ratio“ [41, 53]. However, because SROI analyses are inherently context-specific, their findings may have limited generalizability and comparability [62, 63]. The three included studies conducting a SROI analysis all identified SROI ratios larger than one (between 2.57 and 5.36 : 1 GBP) [49–51]. However, as mentioned in all three studies, the selection of the appropriate financial value for the outcomes is subject to the choice of the researcher, despite being transparent and subject to scrutiny following principle six of the SROI approach [53]. This might introduce a researcher bias, overestimate the social value [50, 51], and would benefit from more stakeholder validation. Adjustments of the social value generated are part of the SROI process to not overestimate the impact of the program. There are four main factors that should be considered: deadweight, attribution, displacement, and drop-off [53]. These are important aspects of a SROI analysis, and the factors are defined based on literature and data from the sample, without, however, being further evaluated in sensitivity analyses [53].

The CBA, as employed in the study by Pretty et al. [48], converts all researcher and/or perspective-chosen benefits into monetary terms, and the total costs are compared to the total benefits, either as a benefit-cost ratio or as net benefit (i.e., difference between benefits and costs). This type of analysis includes measures beyond health-related outcomes. In contrast to a CEA, a cost-benefit evaluation can accommodate different time horizons for costs and benefits. Pretty et al. did not consider health-related outcomes but instead loneliness and LS/H as outcomes [48]. The authors concluded that the economic returns of the examined programs range from 6,000 to 14,000 GBP for one year, and the public benefit–private cost ratio varied between 15.1 and 15.8 : 1 when accounting for all prevented costs and the improvement of life satisfaction. However, besides the limitations mentioned above in the results (e.g., not considering landscape quality), converting benefit outcomes into monetary terms presents challenges. Pretty et al. did not account for potential changes in employment status or other major life events, which might affect the results [48]. Moreover, Pretty et al. monetized improvements in life satisfaction and happiness based on assumptions and prior literature, which may introduce further biases [48].

Finally, the CUA, as conducted by Willis et al. [47], is a widely used approach in the comparative evaluation of medical or health policy interventions [64, 65]. This approach allows to assess the balance between benefits in terms of health outcomes, such as QALYs, and the costs of interventions by calculating the incremental cost-utility ratio (ICUR). The study by Willis et al. [47] included QALYs as a measure of benefit and evaluated the HRQoL of participants before and after the NBSP intervention. The resulting incremental pre-post costs per QALY gained for the NBSP intervention were then compared to the commonly adopted willingness-to-pay threshold of 30,000 GBP per QALY in the UK [47]. Willis et al. [47] concluded that the analyzed NBSP program − 8,600 GBP or 11,400 GBP per QALY gained, depending on the setting considered − can be considered cost-effective. This study was also referenced by Lynch et al. [35] in their systematic review of economic evidence on the costs of improving public health through access to and use of natural environments. The review included a broad range of different economic evaluation methods, with Willis et al. [47] being the only study to conduct a CUA.

Although SROI, CBA, and CUA were all used to evaluate NBSP interventions, differences in their underlying methodologies limit the direct comparability of results across studies. SROI and CBA express outcomes in monetary terms but differ in their valuation approaches and assumptions, while CUA reports outcomes in non-monetary units, i.e., QALYs. As a result, effect estimates and summary metrics (e.g., SROI ratios versus ICURs) cannot be compared quantitatively across evaluation types. Comparability is, therefore, largely limited to the direction of effects, the types of costs and benefits included, and the analytic perspective adopted. These methodological differences should be considered when interpreting findings and drawing conclusions across studies.

At the same time, the included studies indicate a growing interest in the economic evaluation of NBSP, but also highlight the need for methodological advances to strengthen the evidence base. Future evaluations would benefit from appropriate control or comparison groups to improve causal interpretations and reduce the risk of bias inherent in pre-post analyses. Greater clarity of analytic perspectives are also needed. In addition, longer and empirically justified time horizons, combined with sensitivity analyses to explore uncertainty in key assumptions, would be needed to capture the preventive nature of NBSP interventions. The more consistent use of standardized outcome measures, as well as transparent and transferable approaches to valuing non-market benefits, would further enhance comparability and robustness.

In addition to the abovementioned limitations of the individual studies included in our systematic review, these studies exhibited further limitations that need to be considered for the interpretation of the findings. As reflected in the quality assessment using the CHEC list, the interpretation of the economic findings is substantially constrained by methodological limitations in the underlying effectiveness evidence. One significant issue is the sample sizes across the studies, which varied between 59 and 642 people, with a mean sample size of 210. However, four out of the five studies had a sample size smaller than 160 and several evaluations experienced substantial attrition between baseline and follow-up, further weakening confidence in observed outcome changes. Small sample sizes and loss to follow-up limit the statistical power of the analyses and affect the generalizability of the results. As also mentioned before, none of the included studies employed a control or comparison group and relied exclusively on pre-post study designs, which limits causal attribution and increases the risk of bias. The absence of comparators is particularly problematic given that participants often received concurrent services or support, making it difficult to disentangle the specific contribution of NBSP interventions. As participant recruitment was based on self-referral or professional referral rather than random selection in all studies, selection bias cannot be ruled out, particularly given the voluntary nature of participation and the absence of comparator groups. Furthermore, the perspectives adopted in the health economic analyses were not always clearly specified, except for the study by Jones et al. [49], which explicitly adopted a societal perspective. The lack of explicit transparency regarding the perspectives taken in these studies makes it difficult to assess the applicability of the findings across different healthcare systems or policy contexts. Another limitation is that the discounting rate (except for Pretty et al. [48] and Jones et al. [49]) and the cost year (except for Hartfiel et al. [50], and Makanjuola et al. [51]) were not always clearly stated, which can influence the accuracy and comparability of cost estimates. In addition, short or insufficiently justified follow-up periods and heterogeneous, predominantly self-reported outcome measures weaken causal interpretations and, thus, the robustness of the economic findings. These methodological issues, combined with the heterogeneous definitions of NBSP, the diversity of the programs assessed and the variation in outcomes measured, restrict the generalizability and interpretability of the findings. These limitations highlight the need for more rigorous and transparent reporting in future studies to strengthen the evidence base and provide more reliable guidance for policy decision-making.

While loneliness and social isolation are key targets of NBSP interventions, these outcomes should also be seen as intermediate steps towards improving participants’ mental and physical health and well-being. One challenge is that trials and the corresponding intervention duration may not be long enough to capture the long-term health improvement resulting from reduced loneliness. However, limited study durations are common across many medical fields and studies. Often, trials measure surrogate outcomes, and cost-effectiveness analyses integrate these results with evidence from epidemiological studies to extrapolate effects beyond the trial period. This extrapolation can be achieved using decision-analytic models. However, as identified in a review by Rochau et al. [66], evidence on this topic is very scarce, with only two studies overall having conducted a decision-analytic model for social prescribing interventions [67, 68] but not specifically for NBSP interventions.

Our systematic review has strengths and limitations beyond those of the individual studies included in the review. The main strengths of this review include the application of strict screening procedures including independent screening and evaluation by two independent reviewers, the definition of standardized extraction criteria, the search in six different literature databases, and the use of an internationally recognized and well-established checklist for the quality assessment of economic studies [44]. The review has also been registered in PROSPERO (PROSPERO 2021 CRD42021286176), which enhances transparency and supports the reproducibility of the review findings.

Nevertheless, our review also has several limitations. We included only studies published as full-text articles in peer-reviewed journals. Thus, the exclusion of grey literature may have introduced publication bias, as relevant unpublished or non–peer-reviewed evaluations of NBSP interventions were not captured. Additionally, we restricted our search to studies published in English, German, and Spanish after the year 2000, which may have resulted in the omission of potentially relevant literature. We may have missed relevant studies if the authors did not use the terms that we included in our search strategy (as presented in the supplemental materials (Supplemental methods 2). The definition of NBSP and the programs assessed in the studies are heterogeneous, limiting the completeness and generalizability of the findings. Our search criteria excluded social prescribing interventions where nature-based activities were not foregrounded but may have featured as a program component. Finally, as all included studies were conducted in the UK, differences in cost structures and healthcare system may reduce the applicability of these findings to other countries with different systems, thereby limiting the transferability of the health economic results to the context of other countries, settings, and healthcare systems. This highlights the need for further research to conduct similar studies in other countries.

## Conclusion

In our systematic review, we identified five UK-based studies evaluating the health-economic impact of nature-based social prescribing interventions, assessed their quality using the CHEC list, and identified methodological limitations highlighting areas for improvement. The evidence synthesized in our review supports the hypothesis that NBSP initiatives may be associated with positive effects on mental health and well-being, particularly among vulnerable populations. Additionally, several studies suggest potential for reduced utilization of healthcare resources, such as visits to GPs, specialist consultations, and medication use, potentially reducing the financial burden on healthcare systems.

Overall, the studies reported favorable effects of NBSP on health and economic outcomes. However, these results should be interpreted with caution. Several weaknesses were identified, which should be addressed in the future to strengthen the evidence base and better support health policy decision-makers. These weaknesses include small sample sizes and loss to follow-up, absence of control groups and reliance on pre-post study designs, incomplete data on healthcare resources, and a lack of information on the long-term effects of the intervention, all of which constrain causal interpretations and the robustness of economic conclusion. While the findings suggest that NBSP could be a valuable component of the UK NHS strategy, international evidence on this topic remains limited and more rigorous, transparently reported, and methodologically consistent economic evaluations are needed.

## Supplementary Information

Supplementary Material 1.

## Data Availability

The authors confirm that supporting data to the findings of this review are available within this article and the Supporting Information. Additional data that support the findings of this study are available from the corresponding author upon reasonable request.
