# Supplementary material for: Systematic literature review of economic studies on nature-based social prescribing for health improvement
Source: BMC Prim Care. 2026 Mar 14;27:150. doi: 10.1186/s12875-026-03258-w (PMC13101246; doi:10.1186/s12875-026-03258-w)
Supplement: Supplementary file 1 — Supplementary Material 1. [file 12875_2026_3258_MOESM2_ESM.docx]

**Systematic Literature Review of Economic Studies on Nature-Based Social Prescribing for Health Improvement - Supporting Information**

1. **Study selection process**

**Brief summary, details are available in the study registration (https://www.crd.york.ac.uk/prospero/display_record.php?RecordID=286176)**

We are interested in primary studies or interesting reviews (to later identify the primary studies) that include any cost analyses (e.g., only costs or cost-effectiveness or decision analytic model) regarding any nature-based social prescribing (e.g., doctors/health professionals or other organizational representative prescribed/suggested some NBSP) irrespectively of the treatment goal.

Examples of NBSP can be walking in the woods, gardening activities, walking near the river etc. These interventions are mainly used to treat depression, loneliness, anxiety, social isolation etc.

For unifying the screening process, we use the following criteria:

1. Is the article's full text available in English/German/Spanish?

2. Is this a primary, full-text article or a relevant review?

3. Is the intervention a NBSP?

4. Is there a cost or economic evaluation?

**Technical screening procedure**

**We will use the “Research notes” section in EndNote reference management software to make the notes.**

Please be consistent in writing the notes so we can match all screening results.

If the inclusion criteria is fulfilled write “INC”, otherwise write “EXC” and provide one of the exclusion reasons (e.g., for question 3 – “q3noNBSP”). Please note, always use the same explanations and write them as a compound word.

If you are not sure about the inclusion criteria write down the question note, followed by “?” (e.g., for question 3 – “q3noNBSP?”). These studies will further be included in the next step of full-text screening.

If an article does not follow some of the above requirements but may be useful for writing our background/discussion, write down “!” after the “EXC” note. If duplicate write “Dupl”.

Notes to be used (you can add new ones if you need):

INC

EXC q1noLang

EXC q2noPrim

EXC q3noNBSP

EXC q3noNBSP OnlySP

EXC q4noCost

EXC Dupl

1. **Literature review search codes**

Studies were screened after the year of 2000.

1. **Medline accessed via PubMed**

((nature based[Title/Abstract] OR green-solution*[Title/Abstract] OR natural-spac*[Title/Abstract] OR “natural environment”[Title/Abstract] OR “natural environments”[Title/Abstract] OR green-spac*[Title/Abstract] OR greenspace*[Title/Abstract] OR blue-spac*[Title/Abstract] OR open-spac*[Title/Abstract] OR green-infrastructur*[Title/Abstract] OR blue-infrastructur*[Title/Abstract] OR natural-infrastructur*[Title/Abstract] OR nature-contact*[Title/Abstract] OR nature-exposur*[Title/Abstract] OR nature-experienc*[Title/Abstract] natural-therap*[Title/Abstract] OR nature-therap*[Title/Abstract] OR ecotherap*[Title/Abstract] OR wilderness-therap*[Title/Abstract] OR forest-bath*[Title/Abstract] OR gardening*[Title/Abstract] OR horticulture*[Title/Abstract] OR outdoor-activit*[Title/Abstract] OR green-exerci*[Title/Abstract] OR renaturali* [Title/Abstract] OR care-farm*[Title/Abstract] OR social-prescri*[Title/Abstract] OR green-prescri*[Title/Abstract])

AND

("Economics"[Mesh] OR economic*[Title/Abstract] OR Costs and Cost Analysis[Mesh] OR cost*[Title/Abstract] OR cost-effectiv*[Title/Abstract] OR cost-utilit*[Title/Abstract] OR cost-saving*[Title/Abstract] OR cost-mini*[Title/Abstract] OR economic-evaluat*[Title/Abstract] OR cost-benefit*[Title/Abstract] OR benefit-harm*[Title/Abstract] OR cost-impact*[Title/Abstract] OR "return on investment"[Title/Abstract] OR SROI[Title/Abstract] OR social-value[Title/Abstract] OR social-impact[Title/Abstract] OR blended-value[Title/Abstract]))

1. **Embase accessed via Ovid**

(nature based or green-solution* or natural-spac* or green-spac* or greenspace* or blue-spac* or open-spac* or green-infrastructur* or blue-infrastructur* or natural-infrastructur* or nature-contact* or nature-exposur* or nature-experienc* or natural-therap* or nature-therap* or ecotherap* or wilderness-therap* or forest-bath* or gardening* or horticulture* or outdoor-activit* or green-exerci* or renaturali* or care-farm* or social-prescri* or green-prescri*).ti,ab,cl,oa,kw,kf. and

(*health economics/ or *environmental economics/ or economics/ or *"cost of illness"/ or *"cost effectiveness analysis"/ or *"cost of living"/ or *"cost utility analysis"/ or *"health care cost"/ or *"opportunity cost"/ or *"cost benefit analysis"/ or *"program cost effectiveness"/ or *"cost"/ or *"hospital cost"/ or *"nursing cost"/ or *"cost minimization analysis"/ or cost-effectiv*.ti,ab,cl,oa,kw,kf. or cost-utilit*.ti,ab,cl,oa,kw,kf. or cost-saving*.ti,ab,cl,oa,kw,kf. or cost-mini*.ti,ab,cl,oa,kw,kf. or economic-evaluat*.ti,ab,cl,oa,kw,kf. or cost-benefit*.ti,ab,cl,oa,kw,kf. or benefit-harm*.ti,ab,cl,oa,kw,kf. or cost-impact*.ti,ab,cl,oa,kw,kf. or "return on investment".ti,ab,cl,oa,kw,kf. or SROI.ti,ab,cl,oa,kw,kf. or social-value.ti,ab,cl,oa,kw,kf. or social-impact.ti,ab,cl,oa,kw,kf. or blended-value.ti,ab,cl,oa,kw,kf.)

1. **The National Health Service Economic Evaluation Database**

((MeSH DESCRIPTOR Nature EXPLODE ALL TREES) OR (MeSH DESCRIPTOR Relaxation Therapy EXPLODE ALL TREES) OR ((nature OR natural) NEAR2 (based OR spac* OR environment* OR infrastructur* OR exposur* OR contact* OR experienc* OR therap*)) OR ((green OR blue OR open OR wilderness OR forest OR outdoor OR social* OR walk*) NEAR2 (solution* OR spac* OR environment* OR infrastructur* OR exerci* OR therap* OR prescri*)) OR (ecotherap* OR gardening* OR horticulture* OR renaturali* OR farm*))

AND

((MeSH DESCRIPTOR Economics EXPLODE ALL TREES) OR ((cost or economic* or social) NEAR2 (utilit* OR effectiv* OR saving* OR mini* OR evaluat* OR benefit* OR value* OR return OR impact* OR therap*)) OR (benefit-harm* OR economic* OR cost)) FROM 2000 TO 2024

1. **Global Health Cost Effectiveness Analysis Registry**

We screened studies within the “Psychosocial” and “Other” categories.

1. **International Health Technology Assessment Database**

((nature based)[abs] OR (green solution*)[abs] OR (natural spac*)[abs] OR (natural environment*)[abs] OR (green spac*)[abs] OR (greenspace*)[abs] OR (blue spac*)[abs] OR (open spac*)[abs] OR (green infrastructur*)[abs] OR (blue infrastructur*)[abs] OR (natural infrastructur*)[abs] OR (nature contact*)[abs] OR (nature exposur*)[abs] OR (nature experienc*)[abs] OR (natural therap*)[abs] OR (nature therap*)[abs] OR (ecotherap*)[abs] OR (wilderness therap*)[Title/Abstract] OR (forest bath*)[abs] OR (gardening*)[abs] OR (horticulture*)[abs] OR (outdoor activit*)[abs] OR (green exerci*)[abs] OR (renaturali*)[abs] OR (care farm*)[abs] OR (social prescri*)[abs] OR (green prescri*)[abs] OR "Nature"[mh] OR "Environment and Public Health"[mh] OR "Environment"[mh] OR "Horticultural Therapy"[mh] OR "Exercise Therapy"[mh] OR "Social Behavior"[mh] OR "Psychology, Social"[mh] OR "Social Adjustment"[mh] OR "Reinforcement, Social"[mh] OR "Social Integration"[mh] OR "Social Inclusion"[mh] OR "Social Isolation"[mh] OR "Occupational Therapy"[mh] OR "Socioenvironmental Therapy"[mh])

AND

("Economics"[mh] OR (economic*)[abs] OR "Costs and Cost Analysis"[mh] OR (cost*)[abs] OR (cost effectiv*)[abs] OR (cost utilit*)[abs] OR (cost saving*)[abs] OR (cost mini*)[abs] OR (economic evaluat*)[abs] OR (cost benefit*)[abs] OR (benefit harm*)[abs] OR (cost impact*)[abs] OR (return on investment)[abs] OR (SROI)[abs] OR (social value)[abs] OR (social impact)[abs] OR (blended value)[abs])

1. **EconLit accessed via EBSCO**

(((MM "Environment and Public Health") OR (MM "Natural Environment") OR (MM "Natural and Biologically Based Therapies") OR TI(((nature OR natural) N2 (based OR spac* OR infrastructur* OR exposur* OR contact* OR experienc* OR therap*)) OR ((green OR blue OR open OR wilderness OR forest OR outdoor OR social* OR walk*) N2 (solution* OR space OR infrastructur* OR exerci* OR therap* OR prescri*)) OR (ecotherap* OR gardening* OR horticulture* OR renaturali* OR farming*))) OR AB(((nature OR natural) N2 (based OR spac* OR infrastructur* OR exposur* OR contact* OR experienc* OR therap*)) OR ((green OR blue OR open OR wilderness OR forest OR outdoor OR social* OR walk*) N2 (solution* OR space OR infrastructur* OR exerci* OR therap* OR prescri*)) OR (ecotherap* OR gardening* OR horticulture* OR renaturali* OR farming*))))

AND

(((MM "Economics") OR (MM "Costs and Cost Analysis") OR (MM "Economic Aspects of Illness") OR TI (((cost or economic* or social) N2 (utilit* OR effectiv* OR saving* OR mini* OR evaluat* OR benefit* OR value* OR return OR impact*)) OR (benefit-harm*))) OR AB (((cost or economic* or social) N2 (utilit* OR effectiv* OR saving* OR mini* OR evaluat* OR benefit* OR value* OR return OR impact*)) OR (benefit-harm*))))

**Table S1** Assessment of the methodological quality of the included studies based on the Consensus on Health Economic Criteria (CHEC) list (44)

| **CHEC Items** | **Hartfiel (50)** | **Jones (49)** | **Makanjuola (51)** | **Pretty (48)** | **Willis (47)** |
| --- | --- | --- | --- | --- | --- |
| 1. Is the study population clearly described? | yes | yes | yes | no | no |
| 2. Are competing alternatives clearly described? | N/A | N/A | N/A | N/A | N/A |
| 3. Is a well-defined research question posed in answerable form? | yes | yes | yes | no | yes |
| 4. Is the economic study design appropriate to the stated objective? | yes | yes | yes | yes | yes |
| 5. Is the chosen time horizon appropriate to include relevant costs and consequences? | no | yes | no | yes | yes |
| 6. Is the actual perspective chosen appropriate? | N/A | yes | N/A | N/A | yes |
| 7. Are all important and relevant costs for each alternative identified? | yes | yes | yes | no | yes |
| 8. Are all costs measured appropriately in physical units? | yes | yes | yes | yes | yes |
| 9. Are costs valued appropriately? | yes | yes | yes | yes | yes |
| 10. Are all important and relevant outcomes for each alternative identified? | yes | N/A | yes | no | N/A |
| 11. Are all outcomes measured appropriately? | yes | yes | yes | no | yes |
| 12. Are outcomes valued appropriately? | yes | yes | yes | yes | yes |
| 13. Is an incremental analysis of costs and outcomes of alternatives performed? | N/A | N/A | N/A | N/A | N/A |
| 14. Are all future costs and outcomes discounted appropriately? | N/A | yes | N/A | yes | N/A |
| 15. Are all important variables, whose values are uncertain, appropriately subjected to sensitivity analysis? | no | no | no | no | no |
| 16. Do the conclusions follow from the data reported? | yes | yes | yes | yes | yes |
| 17. Does the study discuss the generalizability of the results to other settings and patient/client groups? | yes | no | yes | yes | no |
| 18. Does the article indicate that there is no potential conflict of interest of study researcher(s) and funder(s)? | yes | yes | yes | yes | yes |
| 19. Are ethical and distributional issues discussed appropriately? | no | no | no | no | no |

Note: yes – Item reported; no – Item not reported; N/A – Item not applicable.
